# Soil Respiration of Paddy Soils Were Stimulated by Semiconductor Minerals

**DOI:** 10.3389/fpls.2022.941144

**Published:** 2022-06-27

**Authors:** Yinping Bai, Ling Nan, Qing Wang, Weiqi Wang, Jiangbo Hai, Xiaoya Yu, Qin Cao, Jing Huang, Rongping Zhang, Yunwei Han, Min Yang, Gang Yang

**Affiliations:** ^1^School of Environment and Resource, Southwest University of Science and Technology, Mianyang, China; ^2^School of Life Science and Engineering, Southwest University of Science and Technology, Mianyang, China; ^3^School of Resources and Environmental Engineering, Tianshui Normal University, Tianshui, China; ^4^Key Laboratory of Humid Subtropical Eco-geographical Process, Ministry of Education, Fujian Normal University, Fuzhou, China; ^5^College of Agronomy, Northwest A&F University, Yangling, China; ^6^School of Tourism and Resources Environment, Qiannan Normal University for Nationalities, Duyun, China

**Keywords:** semiconductor minerals, soil respiration, rice cropped area, photocatalysis, soil carbon

## Abstract

Large quantities of semiconductor minerals on soil surfaces have a sensitive photoelectric response. These semiconductor minerals generate photo-electrons and photo-hole pairs that can stimulate soil oxidation–reduction reactions when exposed to sunlight. We speculated that the photocatalysis of semiconductor minerals would affect soil carbon cycles. As the main component of the carbon cycle, soil respiration from paddy soil is often ignored. Five rice cropping areas in China were chosen for soil sampling. Semiconductor minerals were measured, and three main semiconductor minerals including hematile, rutile, and manganosite were identified in the paddy soils. The identified semiconductor minerals consisted of iron, manganese, and titanium oxides. Content of Fe_2_O_3_, TiO_2_, and MnO in the sampled soil was between 4.21–14%, 0.91–2.72%, and 0.02–0.22%, respectively. Most abundant semiconductor mineral was found in the DBDJ rice cropping area in Jilin province, with the highest content of Fe_2_O_3_ of 14%. Soils from the five main rice cropping areas were also identified as having strong photoelectric response characteristics. The highest photoelectric response was found in the DBDJ rice cropping area in Jilin province with a maximum photocurrent density of 0.48 μA/cm^2^. Soil respiration was monitored under both dark and light (3,000 lux light density) conditions. Soil respiration rates in the five regions were (from highest to lowest): DBDJ > XNDJ > XBDJ > HZSJ > HNSJ. Soil respiration was positively correlated with semiconductor mineral content, and soil respiration was higher under the light treatment than the dark treatment in every rice cropping area. This result suggested that soil respiration was stimulated by semiconductor mineral photocatalysis. This analysis provided indirect evidence of the effect semiconductor mineral photocatalysis has on the carbon cycle within paddy soils, while exploring carbon conversion mechanisms that could provide a new perspective on the soil carbon cycle.

## Introduction

Soil is the largest global carbon sink, with approximately 3,000 Pg of organic carbon (C) in deep soil layers (Kochy et al., 2015). Soil respiration [Rs, i.e., carbon dioxide (CO_2_), which effluxes from the soil surface, including microbial respiration and root respiration; Zhou et al., 2016], contributes large amount of C flux between terrestrial ecosystems and the atmosphere. Approximately, 75–98 Pg of C is released into the atmosphere from the soil each year. That level increases exponentially under global climate change (Schlesinger and Andrews, 2000; Bond-Lamberty and Thomson, 2010; Crowther et al., 2016). Increasing Rs profoundly have been affects global carbon cycling in terrestrial ecosystems and enhance positive feedback loops that contribute to climate warming (Crowther et al., 2016; Wang et al., 2019b). Many previous studies have shown that soil warming accelerates carbon dioxide (CO_2_) fluctuation in the atmosphere (Kampman et al., 2012; Valukenko et al., 2017). If the atmospheric temperature increases by one degree Celsius, global soil carbon stocks in surface soil are predicted to fall by 30–203 Pg carbon (Crowther et al., 2016; Melillo et al., 2017). Soil warming has been previously shown to be the main factor controlling Rs levels (Miao et al., 2020; Lu et al., 2022; Yang et al., 2022). Besides soil warming, other factors enhance Rs, including precipitation, increased nitrogen concentration, and phosphorus deposition (Wang et al., 2019a,b; Liu et al., 2021). However, the mechanisms that changing Rs remain primarily unknown. In addition to climate conditions, a recent study found that geochemistry is an essential factor in controlling soil carbon storage capacity. Some researchers have found that metallic oxides increase soil decomposition (Zhang et al., 2018). However, the relationship between metal oxides with the decomposition and transformation of soil organic matter is not yet clear.

Metallic oxides are soil minerals distributed widely throughout the soils (Liu and Liu, 2022). They affect soil organic matter decomposition by promoting adsorbing, catalytic conversion, and other biotic and abiotic processes (Keiluweit et al., 2015). Most metallic oxides are semiconductor minerals that form a thin coating on the earth’s surface, have photoelectric conversion capabilities, and provide a driving force for redox geochemistry on the earth’s surface through photon-to-electron conversions (Lu et al., 2019b). These inorganic minerals inducing photo catalytic processes have been observed in red soil, which could convert extraneous NO under visible light (Gan et al., 2021). Metallic oxides may also directly impact carbon cycles. It was reported that some kind of metallic oxide were used to enhance humus decomposition (Qi et al., 2019). Humus decomposition accelerated the process of carbon export (Kahkonen and Hakulinen, 2011), however, it remains unclear whether such semiconductor enhanced photocatalysis processes affect soil Rs. Paddy soil is typically rich in semiconductor minerals such as iron and manganese oxides, and has visible light photocatalytic properties affecting soil processes directly (Liu and Liu, 2022). Based on above mentioned analyses, we speculated that the photocatalysis of semiconductor minerals in paddy soils would affect soil carbon cycles. Therefore, the rice cropping region in China was chosen for soil sampling to study the relationship between Rs and semiconductor photocatalysis in paddy soils. This study’s objective was to explore whether soil Rs would be positively affected or impeded by semiconductor photocatalysis under sunlight conditions due to enhanced photon to electron conversions.

## Materials and Methods

### Study Site

**Figure 1:**
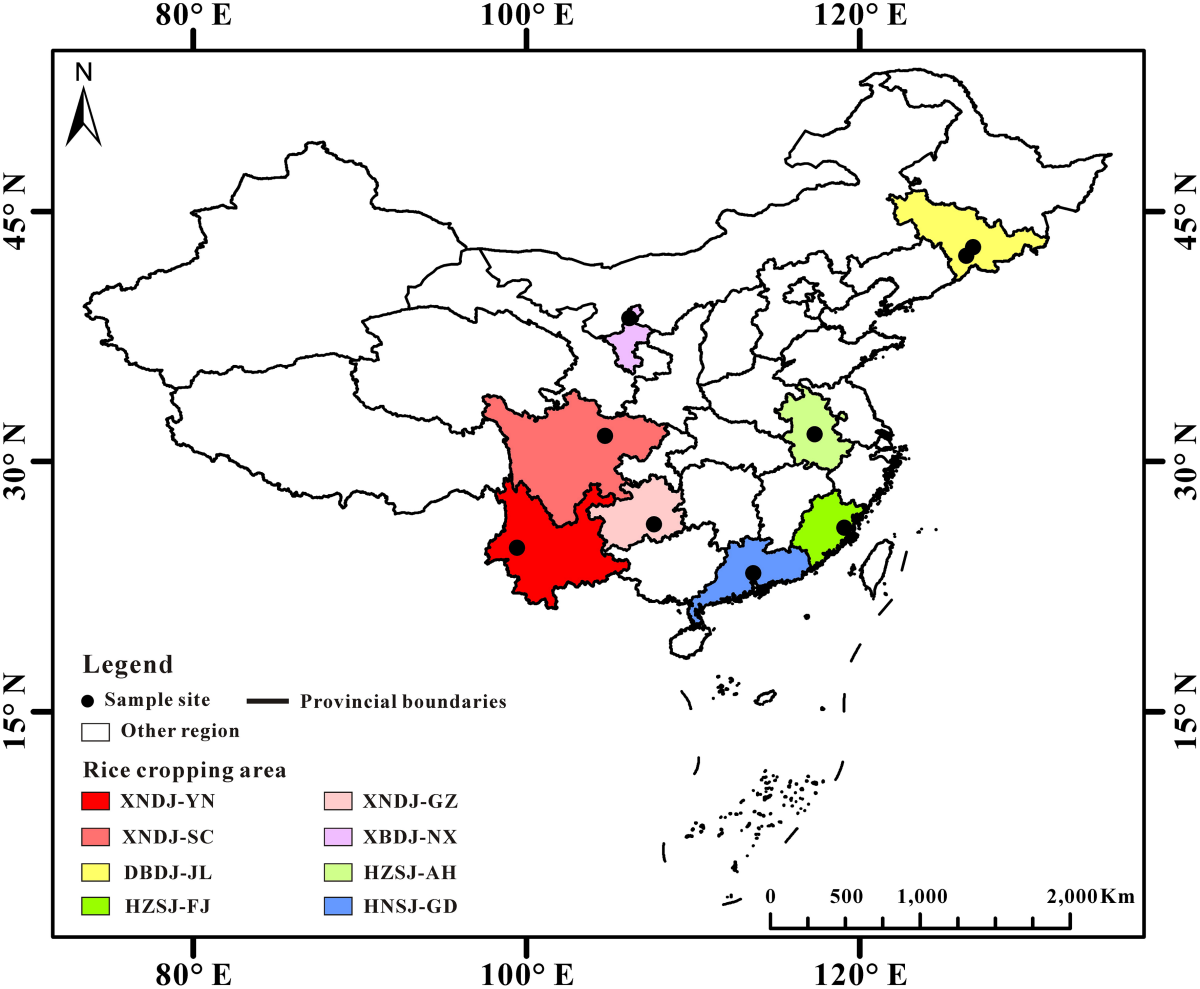
Map of the study sites. Red indicates Yunnan province, Sichuan province, and Guizhou province (part of XNDJ rice cropping area). Yellow indicates Jilin province (part of DBDJ rice cropping area). Green color including Anhui province and Fujian province (part of HZSJ rice cropping area). Blue indicates Guangdong province (part of HNSJ rice cropping area). The five-pointed indicates the location of sample site.

The study sites were located in five major rice cropping areas in China (Figure 1). The sample sites cover the main rice production areas in China. Soils were sampled from double-cropped rice fields in the Guangdong province in Southern China (GD), double-cropped rice fields in Fujian (FJ) and Anhui (AH) province in Center China, single-cropped rice fields in Yunnan (YN) and Guizhou (GZ) province in western China, single-cropped rice fields in Jilin province (JL) in northeastern China, and single-cropped rice fields in the Ningxia (NX) Hui Autonomous Region in Northwestern China.

### Data Collection and Laboratory Analysis

#### Soil Sampling

All soil samples were collected in 2018. Five soil samples were collected at each site from two different layers (0–5 and 5–10 cm deep). The soil samples were divided into two parts. The first fraction was passed through a 2.0 mm mesh sieve and stored at 4°C before measuring soil respiration, total organic carbon (TOC), total organic nitrogen (TON), ammonium nitrogen (NH_3_-N), and nitrate nitrogen (NO_3_-N) levels. The second fraction was air-dried and passed through a 0.15 mm mesh sieve before measuring total carbon (TC) and total nitrogen (TN) levels, and mineral characteristics.

#### Measurement of Soil Physicochemical Parameters

Total carbon and total nitrogen were determined using a total organic carbon analyzer (LIQUIL TOCII, Elementar, Germany) and an automatic azotometer (Kjeltec TM 8400 Analyzer Unit, Foss, Sweden). Moist soil (10 g) was weighed and put into a 250 ml conical bottle, with 50 ml 2 mol/L KCl added and centrifuged for 1 h with the rotation speed of 200 r/min. After extraction and filtration of the supernatant, NH_3_-N and NO_3_-N concentrations were determined using a total organic carbon analyzer (Continuous-Flow Analysis-AA3, SEAL, Germany). The filtered supernatant was then diluted 20 times to determine TOC and TON using a multi N/C 2100 (Analytik Jena, Germany). Soil respiration was determined using a closed culture method in the laboratory. Weigh 25 g of fresh soil and evenly spread it on the bottom of the 500 ml culture flask. Place a 25 ml beaker with 0.1 mol.L^−1^ NaOH solution in the culture flask, and quickly cover and seal the culture flask. Incubate at a constant temperature of 28°C for 24 h to determine the amount of carbon dioxide released. The CO_2_ released from the soil was absorbed by a 0.1 mol.L^−1^ NaOH solution. Then, the NaOH was titrated with standard HCl solution (c = 0.05 mol.L^−1^) to measure the amount of released CO_2_ (Lu, 2002). X-ray fluorescence (XRF) spectrometry was used to determine the most abundant oxides in soil samples. Variations in soil physicochemical characteristics at the different rice cropping sites are shown in Table 1.

**Table 1:** Soil physicochemical characteristics in different rice cropping areas.

Rice cropping area	Sample site	Location	Soil layer cm	pH	Optical band gap	TC %	TN %	TOC mg/kg	TON mg/kg	NH_3_-N mg/kg	NO_3_-N mg/kg
HNSJ	GD	N 113°15′31″ E 23°18′39″	0–5 5–10	6.3 6.4	2.54	1.1 ± 0.02 1.0 ± 0.01	0.16 ± 0.01 0.15 ± 0.02	42.2 ± 2.2 37.7 ± 4.1	14.5 ± 0.4 11.1 ± 0.4	2.9 ± 0.05 3.2 ± 0.10	17.5 ± 0.23 7.8 ± 0.07
HZSJ	FJ	N 119°1′00″ E 26°1′00″	0–5 5–10	6.4 6.3	2.75	1.8 ± 0.01 1.7 ± 0.05	0.22 ± 0.02 0.20 ± 0.01	43.4 ± 1.5 46.9 ± 2.1	27.9 ± 1.0 25.1 ± 1.6	19.8 ± 0.24 14.0 ± 0.04	27.5 ± 0.41 31.8 ± 0.11
HZSJ	AH	N 117°16′31″ E 31°38′11″	0–5 5–10	7.1 7.0	2.57	1.1 ± 0.01 0.7 ± 0.01	0.17 ± 0.01 0.11 ± 0.01	31.9 ± 2.8 28.5 ± 7.2	10.5 ± 0.7 10.1 ± 0.9	2.4 ± 0.05 2.8 ± 0.1	9.2 ± 0.06 5.8 ± 0.02
XNDJ	SC	N 104°42′00″ E 31°32′00″	0–5 5–10	6.6 6.5	2.31	1.6 ± 0.04 1.5 ± 0.01	0.23 ± 0.04 0.21 ± 0.01	42.8 ± 4.3 34.3 ± 1.2	12.5 ± 0.8 12.1 ± 0.4	5.5 ± 0.05 5.4 ± 0.06	5.9 ± 0.05 6.2 ± 0.03
XNDJ	YN	N 99°24′31″ E 24°49′7″	0–5 5–10	6.9 6.9	2.32	3.5 ± 0.01 3.5 ± 0.09	0.25 ± 0.02 0.24 ± 0.02	12.7 ± 1.5 12.3 ± 3.2	4.7 ± 0.2 14.1 ± 1.1	2.5 ± 0.05 2.6 ± 0.10	17.9 ± 0.05 13.3 ± 0.11
XNDJ	GZ	N 107°77′18″ E 26°14′31″	0–5 5–10	6.5 6.4	2.28	3.5 ± 0.01 3.7 ± 0.05	0.41 ± 0.01 0.40 ± 0.02	56.7 ± 1.5 49.7 ± 0.6	29.1 ± 1.1 22.5 ± 1.2	3.1 ± 0.05 3.0 ± 0.88	34.2 ± 0.09 27.8 ± 0.38
DBDJ	JL1	N 126°47′7″ E 42°51′61″	0–5 5–10	7.3 7.3	2.92	2.1 ± 0.01 1.9 ± 0.04	0.25 ± 0.01 0.19 ± 0.02	117.0 ± 5.2 117.0 ± 1.5	214.4 ± 11.6 30.7 ± 0.8	66.5 ± 6.31 24.7 ± 0.05	82.0 ± 2.23 9.3 ± 0.20
DBDJ	JL2	N 126°22′6″ E 42°21′1″	0–5 5–10	7.0 7.1	2.39	5.6 ± 2.7 6.2 ± 0.06	0.49 ± 0.22 0.52 ± 0.01	65.5 ± 2.5 54.5 ± 0.5	20.6 ± 0.7 21.6 ± 0.3	6.9 ± 0.07 9.3 ± 0.02	11.0 ± 0.07 10.3 ± 0.61
XBDJ	NX	N 106°9′18″ E 38°35′30″	0–5 5–10	7.0 7.3	2.70	2.4 ± 0.01 2.1 ± 0.01	0.13 ± 0.01 0.12 ± 0.01	116.0 ± 3.6 121.6 ± 0.7	10.8 ± 0.5 10.3 ± 1.3	2.5 ± 0.04 3.5 ± 0.01	4.7 ± 0.01 4.5 ± 0.01

TC, total carbon; TN, total nitrogen; TOC, total organic carbon; TON, total organic nitrogen; NH_3_-N, ammonium nitrogen; NO_3_-N, nitrate nitrogen; HNSJ, double-cropped rice growing area in Southern China; HZSJ, double-cropped rice growing area in Central China; XNDJ, single-cropped rice growing area at Southwestern China; DBDJ, single-cropped rice growing area in Northeastern China; XBDJ, single-cropped rice growing area in Northwestern; GD, Guangdong province; FJ, Fujian province; AH, Anhui province; SC, Sichuan province; YN, Yunnan province; GZ, Guizhou province; JL1 and JL2, Jilin province; and NX, Ningxia Hui Autonomous Region.

#### Semiconductor Minerals Analysis

The properties of the minerals were determined using X-ray diffraction (XRD). XRD patterns were obtained using an X Pert Pro X-ray diffract meter set to 2.2 kW with a Cu target, continuous scanning mode, working voltage of 60 kV, current of 50 mA, scanning range of 5–80°, and a scanning speed of 5°/min. The mineral phase mass fraction was quantitatively analyzed with the *k*-value method, which uses corundum (Al_2_O_3_) as the universal internal standard. The *K* value on the PDF card is the integral intensity of the strongest peak of the sample divided by the integral intensity of the strongest peak of the corundum after mixing the sample mass with the alumina (corundum) mass fraction in a 1:1 ratio. When corundum is used as an internal standard, the *K* value of the A phase is as follows:


$$K_{Al_{2}O_{3}}^{A}=\frac{K^{A}}{K^{Al_{2}O_{3}}}=\frac{I_{A}}{I_{Al_{2}O_{3}}}$$

According to the “adiabatic method,” if there are *n* phases in the system, the mass fraction of the *X* phase is:


$$W_{x}=\frac{I_{Xi}}{K_{A}^{X}\overset{N}{\underset{i=A}{\sum }}\frac{I_{i}}{K_{A}^{i}}}$$

#### Electrochemical Characterization

A soil electrode was used as the working electrode. To prepare the working electrode, 10 mg soil, 1,250 μl, 95% ethanol, and 1,250 μl of deionized water were mixed and were ultrasound for 30 min. Then, 100 μl of 5% naphthol (mass fraction) was added, and the mixture was ultrasound for another 20 min. About 200 μl of ultrasonic solution coating was applied to a 20 mm × 20 mm × 1.1 mm FTO (SnO_2_: F) conductive glass, and the film area (10 mm × 20 mm) with even dispersal. After the film was formed, it was dried for 4 h in an oven at 40°C to obtain the soil electrode. A saturated calomel was used as the reference electrode, and a platinum wire was used as the counter electrode. KH_2_PO_4_ solution was added to a diaphragm three-electrode electrolytic cell. The pH of the solution was 5.33. The electrochemical workstation (CHI760E) was used and the applied voltage was set at 0.3 V to obtain the I-T curve. The light source for photocurrent was a 100 mW/cm^2^ Xenon lamp with 50 s interval switch.

#### Band Gap Analysis

BaSO_4_ was used as the reference sample, and the film sample was prepared by the integrating sphere method. The full wavelength (200–2,500 nm) diffuse reflectance spectrum was obtained by scanning a solid ultraviolet absorption spectrometer (Soildspec-3700). The semiconductor band gap width was calculated with the following equation using the Tauc plot method, taking *hv* as the abscissa and (*αhv*)^2^ as the ordinate.


$${\left(\alpha hv\right)}^{2}=K\left(hv-E_{g}\right)$$

where *α*: light absorption coefficient; *h*: Planck constant; *v*: frequency; *K*: constant; and *E_g_*: band gap width.

### Data Analysis

Mean soil physical and chemical properties were calculated by averaging the data from each site. One-way ANOVAs were used to assess the effect of planting location on soil respiration (Duncan’s test was used in the ANOVA analysis). Linear regression was used to assess the effect of semiconductor minerals on soil respiration. All sample data were confirmed for normal distribution before ANOVA analysis. SPSS 20.0 for Windows (SPSS Inc., Chicago, IL, United States) was used for all statistical analysis. Graphs were drawn using Sigmaplot 10.0. Differences were considered statistically significant when the value of *p* was lower than the alpha value (*α* = 0.05).

## Results

### Soil Physical and Chemical Properties

The concentrations of TC and TN decreased as soil depth increased in most rice cropping areas, except for the TC in XNDJ and TC and TN in the DBDJ rice cropping area within Jilin province (Table 1). The TOC concentration range was from 12.3 to 121.6 mg/kg, and the TON concentration range was from 4.7 to 214.4 mg/kg. The TOC and TON concentrations of the soils sampled in Jilin province were much higher than it in any other province. The highest concentrations of NH_3_ and NO_3_ (66.5 and 82.0 mg/kg) were also found in Jilin province (Table 1).

**Figure 2:**
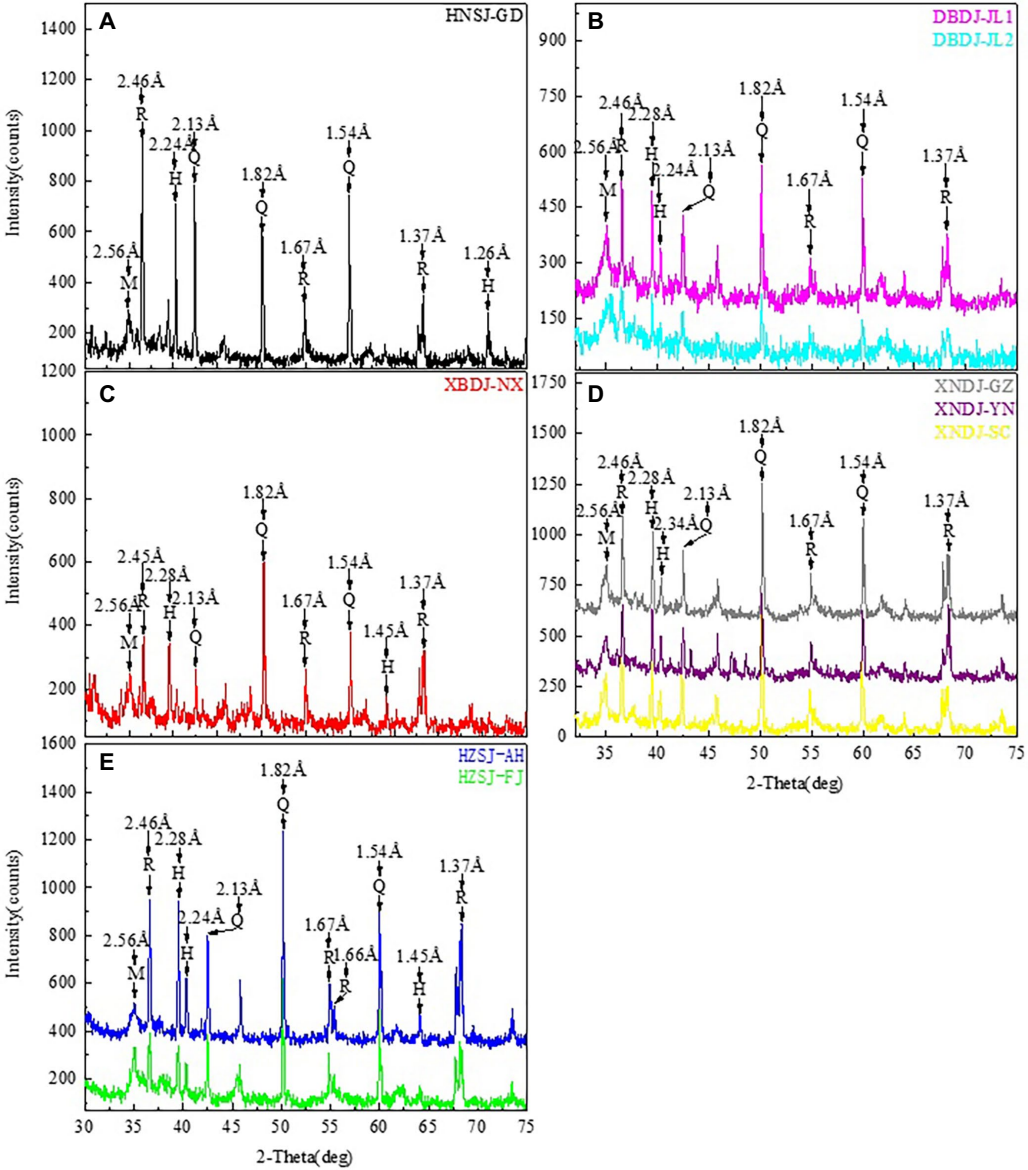
Results of XRD analysis of five different rice soils. **(A–E)** XRD patterns of the 0–5 cm soil layer in HNSJ-GD, DBDJ-JL1, DBDJ-JL2, XBDJ-NX, XNDJ-GZ, XNDJ-YN, XNDJ-SC, HZSJ-AH, and HZSJ-FJ. H, Hematite; R, Rutile; M, Manganosite; and Q, Quartz. HNSJ-GD, double-cropped rice growing area in Guangdong province, Southern China; DBDJ-JL1 and DBDJ-JL2, single-cropped rice growing area in Jilin province, Northeastern China; XBDJ-NX, single-cropped rice growing area in Ningxia Hui Autonomous Region, Northwestern; XNDJ-GD, single-cropped rice growing area at Guangdong province, Southwestern China; XNDJ-YN and XNDJ-SC, single-cropped rice growing area at Yunnan province and Sichuan province, Southwestern China; and HZSJ-AH and HZSJ-FJ, double-cropped rice growing area in Fujian province and Anhui province, Central China.

The oxides identified through XRF analysis included SiO_2_, Al_2_O_3_, Fe_2_O_3_, K_2_O, CaO, TiO_2_, MgO, MnO, P_2_O_5,_ BaO, and Rb_2_O. SiO_2_ was the most abundant oxide with its content varying between 56.01 and 77.91 wt % (Table 2). The content of Al_2_O_3_ ranged between 13.99 and 28.01 wt %. The major semiconductor minerals identified in the five sites were hematile, rutile, and manganosite (Table 3; Figure 2). Fe_2_O_3_, TiO_2_, and MnO Levels were the highest in the DBDJ rice cropping area in Jilin province (Table 2).

**Table 2:** Oxide distributions in soils at different rice growing areas.

Rice cropping area	Sample site	Soil layer	SiO_2_	Al_2_O_3_	Fe_2_O_3_	K_2_O	TiO_2_	CaO	P_2_O_5_	SO_3_	MgO	BaO	MnO	Rb_2_O
HNSJ	GD	0–5	62.58	26.86	4.26	3.6	1.06	0.56	0.49	0.18	0.14	0.05	0.03	0.02
HNSJ	GD	5–10	61.04	28.01	4.4	3.72	1.03	0.6	0.41	0.16	0.13	0.06	0.02	0.02
HZSJ	FJ	0–5	63.16	24.27	5.96	3.27	0.97	0.65	0.61	0.16	0.34	0.07	0.08	0.02
HZSJ	FJ	5–10	62.8	24.46	6.04	3.39	0.99	0.63	0.66	0.15	0.41	0.06	0.08	0.02
HZSJ	AH	0–5	77.12	14.43	4.26	1.91	0.99	0.77	0.13	0.11	/	0.06	0.06	0.01
HZSJ	AH	5–10	77.91	13.99	4.21	1.83	0.95	0.77	0.07	0.05	/	0.05	0.09	0.01
XNDJ	SC	0–5	70.09	18.31	6.33	3.1	0.91	0.79	0.16	0.11	/	0.07	0.05	0.02
XNDJ	SC	5–10	67.74	18.91	7.52	3.38	1.08	0.94	0.11	0.05	/	0.09	0.06	0.02
XNDJ	YN	0–5	57.52	20.44	8.21	2.91	1.17	7.72	0.56	0.21	0.81	0.06	0.15	0.02
XNDJ	YN	5–10	57.5	20.39	8.22	2.91	1.21	8.18	0.54	0.2	0.33	0.06	0.16	0.02
XNDJ	GZ	0–5	67.24	19.73	6.22	3.74	1.41	0.81	0.3	0.27	0.05	0.06	0.02	0.02
XNDJ	GZ	5–10	66.16	20.1	6.36	3.9	1.38	0.76	0.3	0.26	0.42	0.05	0.02	0.02
DBDJ	JL1	0–5	65.96	18.81	6.08	3.34	0.93	2.77	0.43	0.36	0.45	0.08	0.08	0.02
DBDJ	JL1	5–10	66.41	18.76	6.11	3.13	0.93	3.04	0.21	0.12	0.48	0.07	0.09	0.02
DBDJ	JL2	0–5	57.00	21.26	13.46	1.66	2.66	2.64	0.53	0.3	0.03	0.11	0.19	0.01
DBDJ	JL2	5–10	57.44	19.68	14	1.43	2.72	2.89	0.21	0.19	0.06	0.11	0.22	0.01
XBDJ	NX	0–5	56.01	15.35	5.24	3.05	0.69	12.43	0.36	0.39	4.99	0.09	0.07	0.01
XBDJ	NX	5–10	56.77	14.8	4.88	3.39	0.67	12.22	0.33	0.23	5.25	0.08	0.07	0.01

Oxide levels were measured using XRF technology. HNSJ, double-cropped rice growing area in Southern China; HZSJ, double-cropped rice growing area in Central China; XNDJ, single-cropped rice growing area at Southwestern China; DBDJ, single-cropped rice growing area in Northeastern China; XBDJ, single-cropped rice growing area in Northwestern; GD, Guangdong province; FJ, Fujian province; AH, Anhui province; SC, Sichuan province; YN, Yunnan province; GZ, Guizhou province; JL1 and JL2, Jilin province; and NX, Ningxia Hui Autonomous Region.

**Table 3:** Phase mass fractions of minerals collected from five different rice soils.

Rice cropping area	Sample site	Soil layer	Rutile	Hematite	Manganosite	Others
HNSJ	GD	0–5 cm	3.59	0.12	3.11	93.18
HZSJ	FJ	0–5 cm	0.96	1.31	5.07	92.66
HZSJ	AH	0–5 cm	11.39	0.78	1.43	86.40
XNDJ	SC	0–5 cm	5.56	0.16	3.12	91.16
XNDJ	YN	0–5 cm	1.61	0.96	3.62	93.81
XNDJ	GZ	0–5 cm	0.86	0.55	4.70	93.89
DBDJ	JL1	0–5 cm	19.88	5.44	0.36	74.32
DBDJ	JL2	0–5 cm	16.89	1.72	3.18	78.22
XBDJ	NX	0–5 cm	10.23	1.50	2.57	85.71

HNSJ, double-cropped rice growing area in Southern China; HZSJ, double-cropped rice growing area in Central China; XNDJ, single-cropped rice growing area at Southwestern China; DBDJ, single-cropped rice growing area in Northeastern China; XBDJ, single-cropped rice growing area in Northwestern; GD, Guangdong province; FJ, Fujian province; AH, Anhui province; SC, Sichuan province; YN, Yunnan province; GZ, Guizhou province; JL1 and JL2, Jilin province; and NX, Ningxia Hui Autonomous Region.

### Photochemical Properties of the Soil

**Figure 3:**
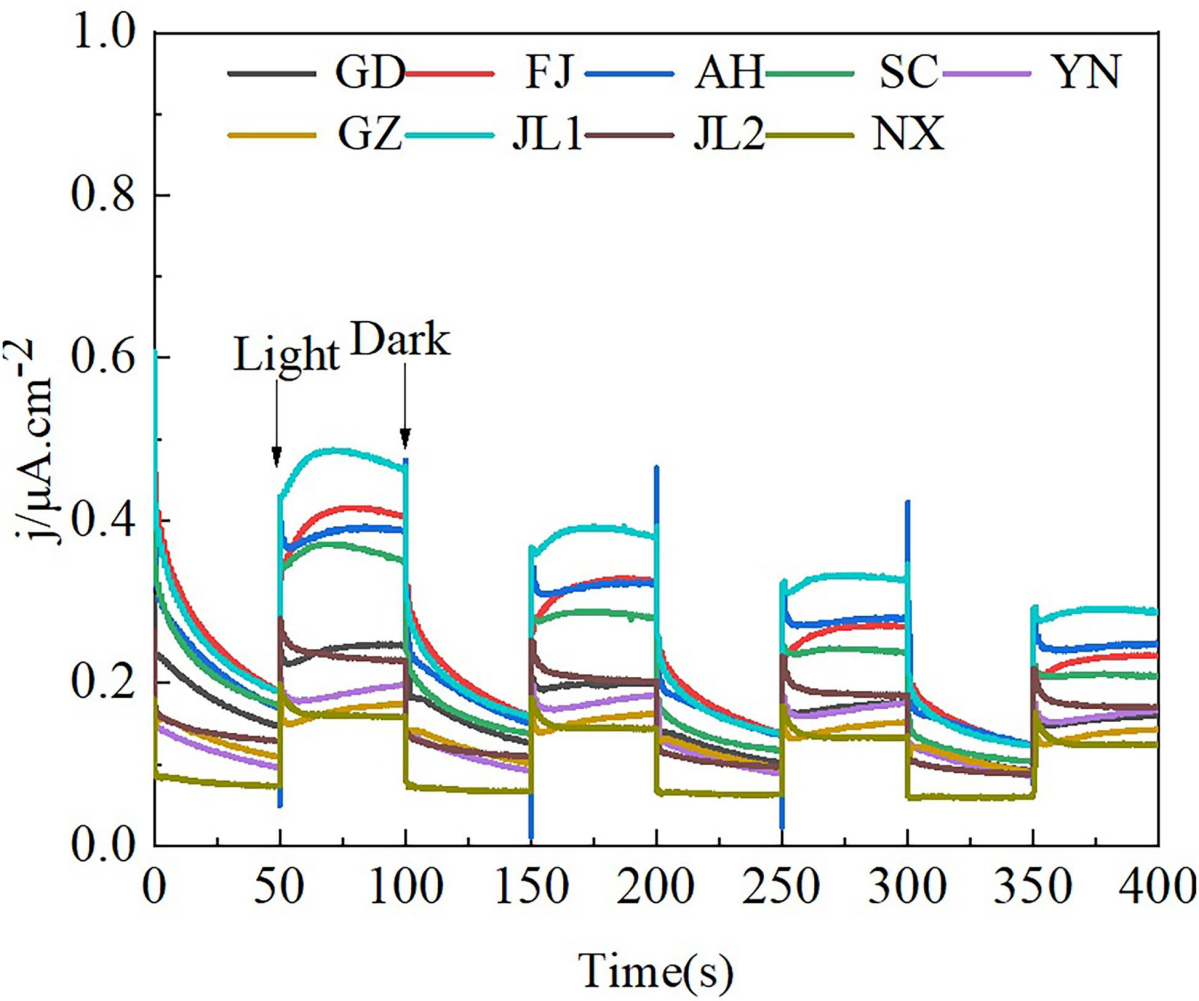
The photocurrent responses of the soil collected from five different rice growing areas. GD, Guangdong province; FJ, Fujian province; AH, Anhui province; SC, Sichuan province; YN, Yunnan province; GZ, Guizhou province; JL1 and JL2, Jilin province; and NX, Ningxia Hui Autonomous Region.

The prepared samples photo responses to intermittent irradiation with visible light at the bias potential of 0.3 V vs.SCE (Figure 3). The maximum photocurrent density of JL1 was 0.48 μA/cm^2^ under illumination. Under dark conditions, the photocurrent density of FJ reached 0.46 μA/cm^2^ which was the highest value observed. When the light and dark period began, the curves rose and fell sharply, indicating that the photoelectric catalysts in the soil produced electrons and holes that recombined and separated quickly. All the samples showed good reproducibility and stability under bias potential. According to UV/Vis diffuse reflectance, the band gap width ranges from 2.28 to 2.92 eV.

### Soil Respiration and Its Relationship With Soil Semiconductors

**Figure 4:**
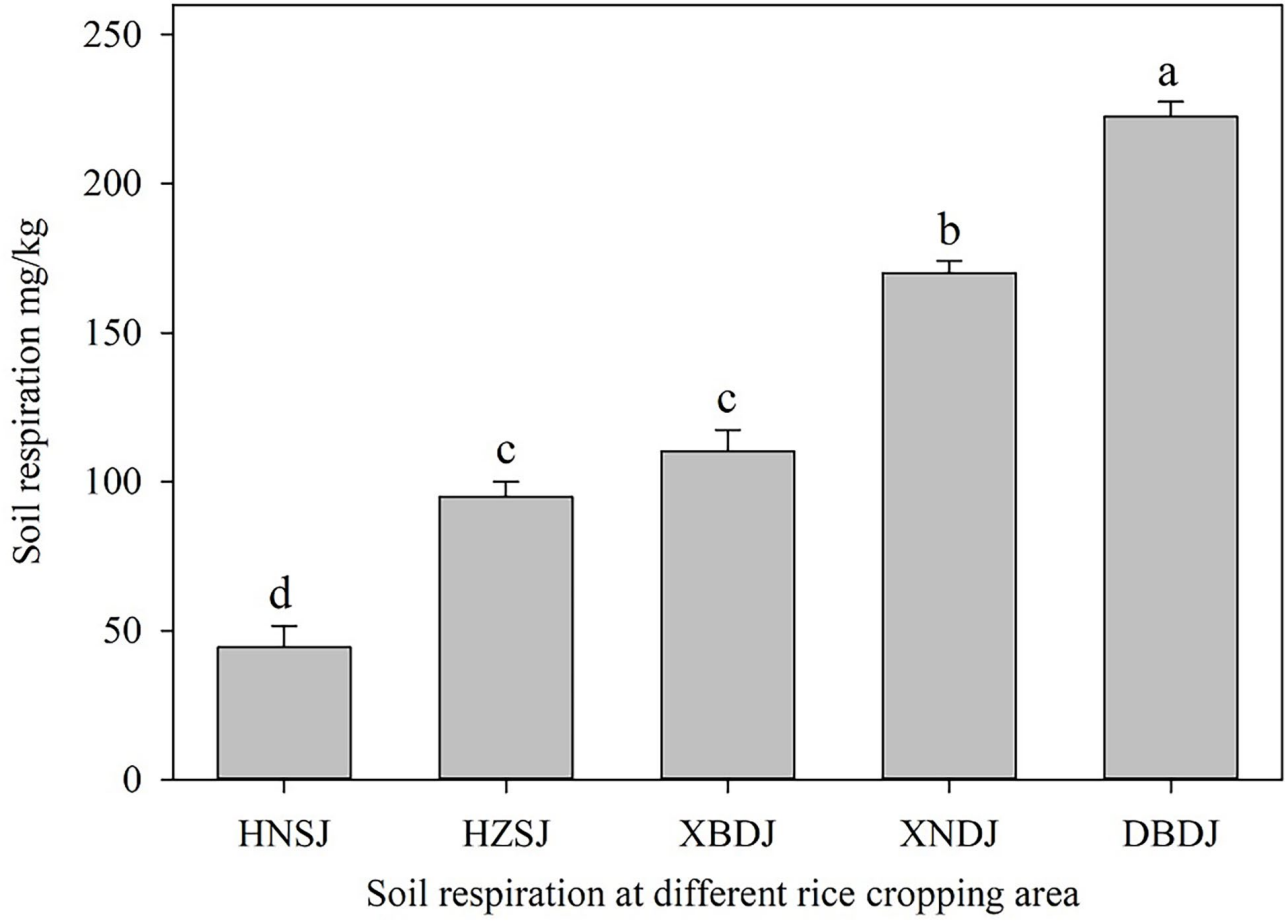
Soil respiration levels (mean ± SE) in five different rice growing areas. Significant differences are indicated by different letters over the error bars (*p* < 0.05).

**Figure 5:**
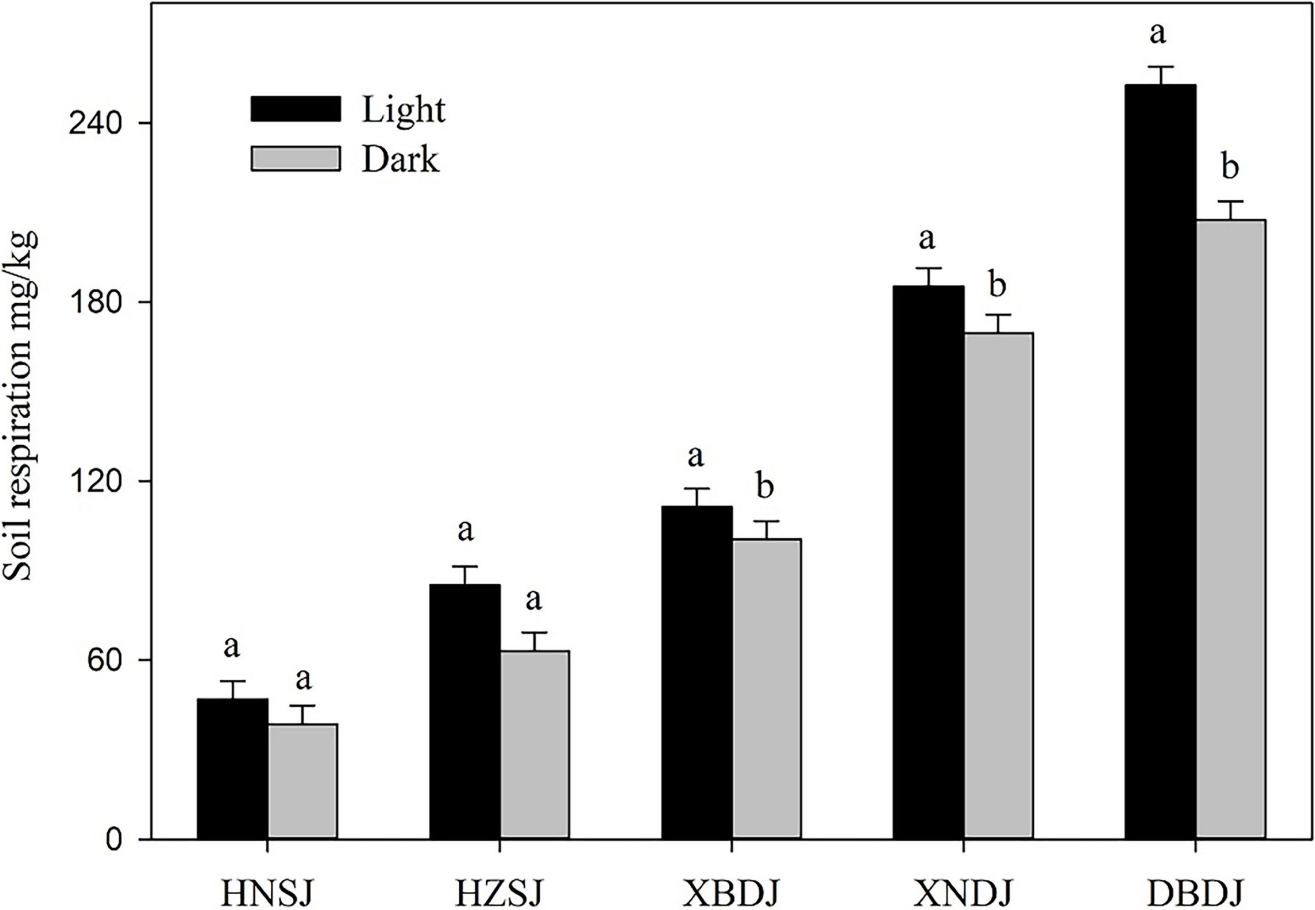
Soil respiration levels (mean ± SE) in different rice growing areas under either dark or light treatments. Significant differences are indicated by different letters over the error bars (*p* < 0.05).

**Figure 6:**
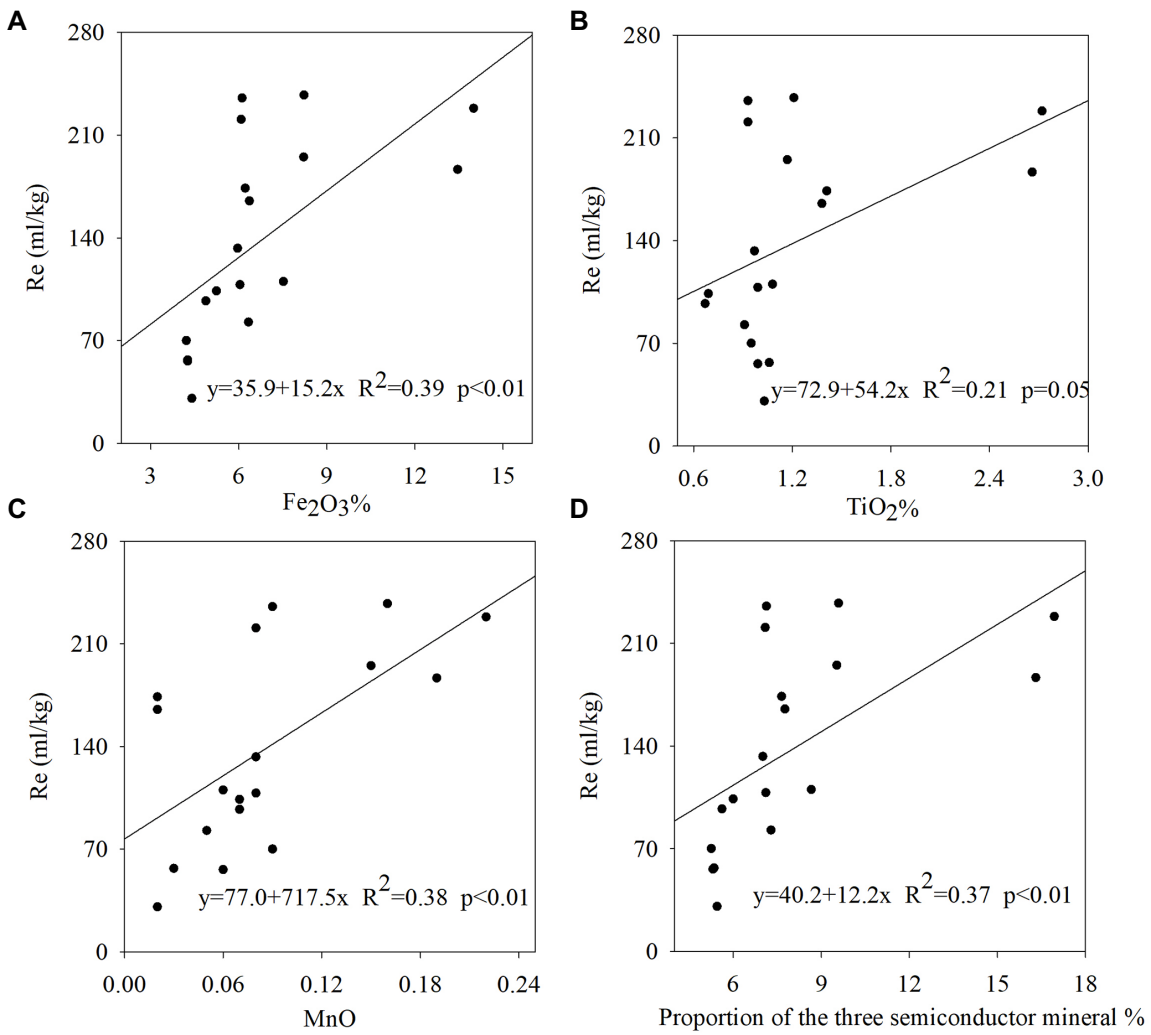
Scatter plots and fitted regression lines for **(A)** soil respiration with Fe_2_O_3_%; **(B)** soil respiration with TiO_2_%; **(C)** soil respiration with MnO%; and **(D)** soil respiration with proportion of the three semiconductor mineral.

In this study, soil respiration significantly differed among the rice cropping area (Figure 4). The highest soil respiration level found in the DBDJ rice cropping area was five times higher than in the HNSJ rice cropping area. From highest to lowest, measured soil respiration ranks at the sample sites were as follows: DBDJ > XNDJ > XBDJ > HZSJ > HNSJ. Soil respiration was significantly higher in the single cropping rice area was in the double cropping rice area (Figure 4). Illumination increased soil respiration levels in all the rice cropping areas. The influence of light was significant in the single cropping rice area (Figure 5). The soil respiration was 10.7, 9.2, and 21.7% higher in light treatment than that in dark treatment. Fe_2_O_3_, TiO_2_, MnO_2_, and the total concentration of these three oxides were significantly positively related to soil respiration. The *R*^2^ value of this positive correlation was greater for Fe_2_O_3_ and MnO than for TiO_2_ (Figure 6).

## Discussion

### Semiconductor Minerals in Different Rice Cropping Area

Ferromanganese oxide as the main semiconductor minerals, which widely developed on soil surfaces and directly exposed under sunlight and impact on ecosystem process (Lu et al., 2019a). In this study, semiconductors like hematite, rutile, and manganosite were found in rice paddy soils throughout China. Semiconductor minerals have important environmental properties and is often used to study their adsorption effect on Pollutants (Ma et al., 2018; Wu et al., 2021). Or using as an indicator for forecasting climate changes (Long et al., 2016). In recent years, the photocatalytic properties of semiconductor mineral have gradually attracted the attention of scientists (Xu et al., 2020). In this study, semiconductor minerals were found in high abundance at the single-cropped rice growing area in Jilin province, Northeastern China. And the lower abundance of semiconductor mineral was found in Southwestern and Southern China. The trends of semiconductor distribution in paddy soil probably attributed to China’s surface annual sunshine duration because semiconductor minerals is always developed on the surface sides of rock and soils, which direct correspondence with sunlight exposure (Li and He, 2010; Lu et al., 2019a).

### Semiconductor Minerals and Soil Respiration

When sunlight hits a semiconductor, photons with energy greater than the forbidden bandwidth can excite the electrons (e^−^) in the valence band to the conduction band to leave a positively charged hole (h^+^). Electrons and electron holes direct contact with oxygen and water molecules producing reactive oxygen species and hydroxyl radicals, stimulating a series of redox reactions (Gan et al., 2021; Li et al., 2021). Semiconductor mineral photocatalysis mediated redox reactions are widely used in solution systems (Luo et al., 2017), but their mechanisms within a soil system remain ambiguous. However, as the most basal process of a soil system, we posit that soil respiration must be affected by semiconductor mineral photocatalysis. In this study, we found the concentration of Fe_2_O_3_, TiO_2_, MnO, and the sum of these three oxides were positively correlated with Rs. Fe_2_O_3_ has been identified as one of the most common iron oxides in soil (Gu et al., 1994). Congruently, it has also been reported that approximately 21.5% of the organic carbon in marine sediments are bound to iron oxides (Lalonde et al., 2012). Organic matter combined with iron oxides have been prone to photocatalytic interfacial reactions and accelerated decomposition of organic matter (Zhang et al., 2019; Chen et al., 2020). TiO_2_, one of the most common metallic oxides in soil, was also found to stimulate soil mineralization and accelerate soil organic carbon loss (Fan et al., 2020). As shown in Figures 3, 5, our data are consistent with the above mentioned results. Our study observed higher photocurrent and soil respiration in illuminated conditions, indicating that electron–hole pairs generated from the soil surface had a positive effect on soil respiration. Therefore, we can postulate that organic carbon is more easily decomposed in the presence of semiconductor minerals, which, inevitably, accelerate soil respiration rates. Based on the available evidence, we hypothesized that the photocatalytic properties of semiconductor minerals are what stimulated the decomposition of organic matter. Previous research has also produced results that are consistent with the data obtained in this current study (Pan et al., 2020). In this study, similar results were recorded in paddy soils of five different regions in China. These observations suggested that a large quantity of semiconductor minerals in surface soils dramatically affected soil respiration in the presence of light. A possible mechanism was that soil particles from the samples contained many metallic oxides and other semiconductor minerals. When sunlight hits these semiconductor minerals, an electron–hole pair with strong oxidation–reduction abilities was excited (Liu et al., 2019; Gan et al., 2021). Our results showed that photo-electrons and photo-holes were separated within a certain period, and direct contact with oxygen and water molecules produced reactive oxygen species and hydroxyl radicals resulting in the production of effective photoelectrons that initiated a series of redox reactions that transmitted energy through the electron transport chain (Fan et al., 2016). Ultimately, this process affects all aspects of soil respiration.

### The Implication of Semiconductor Minerals on Carbon Cycles

On the soil surface, the interaction between light and minerals occurs all the time. It has been reported that 1 m^2^ of rock varnish-covered land surface can produce 2.23 × 10^16^ photoelectrons per second (Lu et al., 2019b), and the photoelectrons produced by these semiconducting minerals rapidly affect organic degradation and gradually impact on soil respiration (Xu et al., 2020). This research provides a new perspective for soil carbon cycle. However, this study ignored the role of microorganisms and the influences of lower valence metals. Previous study has shown that microorganisms are also influenced by the photocatalytic effect of semiconductor minerals (Lu et al., 2012). The evolutionary process of microbial community structure even influenced by photoelectron from semiconducting minerals (Ren et al., 2020). Therefore, future research in this field should examine the effects of semiconductor mineral photocatalysis on soil respiration, while also considering the role of microorganisms play in this process.

## Data Availability Statement

The original contributions presented in the study are included in the article/supplementary material; further inquiries can be directed to the corresponding authors.

## Author Contributions

GY, YB, and QW contributed to conception and design of the experiment. LN, WW, XY, YH, and MY carried out the collection of soil samples. YB analyzed all data, wrote the first draft of the manuscript, and revised it. JHu, RZ, JHa, and QC discussed the first draft. In addition, the natural science fund project applied by GY and QW provided financial support for this research. All authors contributed to the article and approved the submitted version.

## Funding

This study was financially supported by the National Natural Science Foundation of China (42077038) and the Sichuan Science and Technology Program (2020YFS0020).

## Conflict of Interest

The authors declare that the research was conducted in the absence of any commercial or financial relationships that could be construed as a potential conflict of interest.

## Publisher’s Note

All claims expressed in this article are solely those of the authors and do not necessarily represent those of their affiliated organizations, or those of the publisher, the editors and the reviewers. Any product that may be evaluated in this article, or claim that may be made by its manufacturer, is not guaranteed or endorsed by the publisher.

## Abbreviations

HNSJ: Double-cropped rice growing area in Southern China
HZSJ: Double-cropped rice growing area in Central China
XNDJ: Single-cropped rice growing area at Southwestern China
DBDJ: Single-cropped rice growing area in Northeastern China
XBDJ: Single-cropped rice growing area in Northwestern
TC: Total carbon
TN: Total nitrogen
TOC: Total organic carbon
TON: Total organic nitrogen
NH3-N: Ammonium nitrogen
NO3-N: Nitrate nitrogen
GD: Guangdong province
FJ: Fujian province
AH: Anhui province
SC: Sichuan province
YN: Yunnan province
GZ: Guizhou province
JL1 and JL2: Jilin province
NX: Ningxia Hui Autonomous Region
